# IgA Responses Following Recurrent Influenza Virus Vaccination

**DOI:** 10.3389/fimmu.2020.00902

**Published:** 2020-05-19

**Authors:** Rodrigo B. Abreu, Emily F. Clutter, Sara Attari, Giuseppe A. Sautto, Ted M. Ross

**Affiliations:** ^1^Center for Vaccines and Immunology, University of Georgia, Athens, GA, United States; ^2^Department of Infectious Diseases, University of Georgia, Athens, GA, United States

**Keywords:** IgA, influenza, antibody, vaccination, B-cell

## Abstract

Influenza is a highly contagious viral respiratory disease that affects millions of people worldwide each year. Annual vaccination is recommended by the World Health Organization to reduce influenza severity and limit transmission through elicitation of antibodies targeting mainly the hemagglutinin glycoprotein of the influenza virus. Antibodies elicited by current seasonal influenza vaccines are predominantly strain-specific. However, continuous antigenic drift by circulating influenza viruses facilitates escape from pre-existing antibodies requiring frequent reformulation of the seasonal influenza vaccine. Traditionally, immunological responses to influenza vaccination have been largely focused on IgG antibodies, with almost complete disregard of other isotypes. In this report, young adults (18–34 years old) and elderly (65–85 years old) subjects were administered the split inactivated influenza vaccine for 3 consecutive seasons and their serological IgA and IgG responses were profiled. Moreover, correlation analysis showed a positive relationship between vaccine-induced IgA antibody titers and traditional immunological endpoints, exposing vaccine-induced IgA antibodies as an important novel immune correlate during influenza vaccination.

## Introduction

Since the great influenza pandemic of 1918 (Spanish flu), we have struggled to prevent influenza virus infection and transmission. From 2009 to 2019, over 100 million people were infected with the H1N1 swine pandemic virus leading to ~1 million hospitalization and 75,000 deaths (1). During typical influenza seasons, both H1N1 and H3N2 influenza A viruses (IAV) and influenza B viruses (IBV) cause disease morbidity and mortality in the human population (2). Seasonal IAV and IBV co-circulate worldwide in the human population with unpredictable patterns. Consequently, the first influenza monovalent vaccines developed in the 1930s were quickly updated in the 1940s to include an influenza B strain and later to a trivalent formulation with a second IAV strain (3). In this century, the trivalent inactivated vaccine (TIV) was again updated to a quadrivalent (QIV) formulation with inclusion of a second IBV strain to cover the independently evolving IBV lineages (4–6).

Influenza virus infection generates strong and long-lasting immunity, but continuous antigenic evolution of the hemagglutinin (HA) and neuraminidase (NA) surface proteins allows for evasion of pre-existing immunity by drifted strains. Similarly, influenza virus vaccination can transiently induce strain-specific antibodies, but fails to protect against antigenically drifted strains, requiring yearly strain updates to the vaccine (7, 8). Nonetheless, yearly vaccination is still the most effective strategy to prevent and control influenza infection (9).

Recent epidemiological models suggest that virtually everyone in the developed world experiences their first influenza infection by the age of five (10). Moreover, early-life exposure to influenza greatly shapes the immune response to subsequent infections and vaccination through a phenomenon known as immune imprinting or original antigenic sin (11).

Despite our reductive view of vaccine-elicited protection, oversimplified to a couple of clinical and serological endpoints, the immune response to influenza vaccination is a complex network of cellular signals and responses strongly dependent on the subject's past immunological experience (12). First exposure to influenza virus elicits a strong humoral response mainly targeting the viral surface proteins HA and NA (13), ideally driven by balanced pro- and anti-inflammatory signals that lead to viral clearance with minimal tissue damage (14, 15).

Sterilizing immunity in absence of inflammation is promoted by serum neutralizing antibodies against the HA receptor binding site (RBS) (12). Antibodies that bind to this location can prevent viral adhesion and internalization to target cells. To date, serological inhibition of erythrocyte hemagglutination by influenza virus remains the gold standard assay to measure HA-receptor blocking antibodies and evaluate vaccine elicited protection (16). Similarly, serological levels of HA-reactive IgG antibodies are correlated with reduced viral shedding and ameliorated disease (17–19).

Mechanistically, mucosal immunity driven by IgA antibodies and tissue resident memory B- and T-cells is the major contributor for influenza virus protection (12). Furthermore, unadjuvanted inactivated vaccines fail to generate strong T cell-dependent responses (20) and therefore rely on the recall of pre-existing immunity, which is extremely diverse in the human population (21, 22). Nonetheless, the impact of vaccination on the human IgA response and mucosal immunity to influenza viruses is technically challenging, evasive and as so generally overlooked. Recently, Iversen et al. reported that the gut mucosal and serological IgA repertoires of celiac patients share strong clonal overlap despite originating from different plasma cell compartments (23). Moreover, a recent transcriptomic analysis of serological IgA plasmablasts following influenza vaccination seems to indicate a common shared IgA-repertoire between serum and the lung mucosa (24). Contrastingly, mucosal and serological IgG repertoires share lower clonal relatedness than those of IgA subtype (23).

Current influenza virus vaccines provide limited protection, even in well-matched years (25), with particularly low effectiveness in high-risk populations, e.g., young children and elderly (18, 26). Current immunological correlates poorly portray the complex immune response in these populations following influenza virus vaccination. Here, we longitudinally tracked serological changes in vaccine-specific IgA and IgG antibody levels in young adult and elderly subjects following influenza virus vaccination in three consecutive years. Additionally, since the H3N2 IAV vaccine component was updated each season during the course of the study, we further compared vaccine-induced IgA and IgG serological responses against the H1N1 IAV component that was not updated vs. an updated drifting H3N2 IAV antigen. Overall, the relationship between vaccine-specific IgA antibody titers and other immune correlates of protection was evaluated.

## Materials and Methods

### Study Approval

The study procedures, informed consent, and data collection documents were reviewed and approved by the Western Review Board and the Institutional Review Boards of the University of Pittsburgh. The funding source had no role in sample collection nor decision to submit the paper for publication.

### Subjects

**Figure 1:**
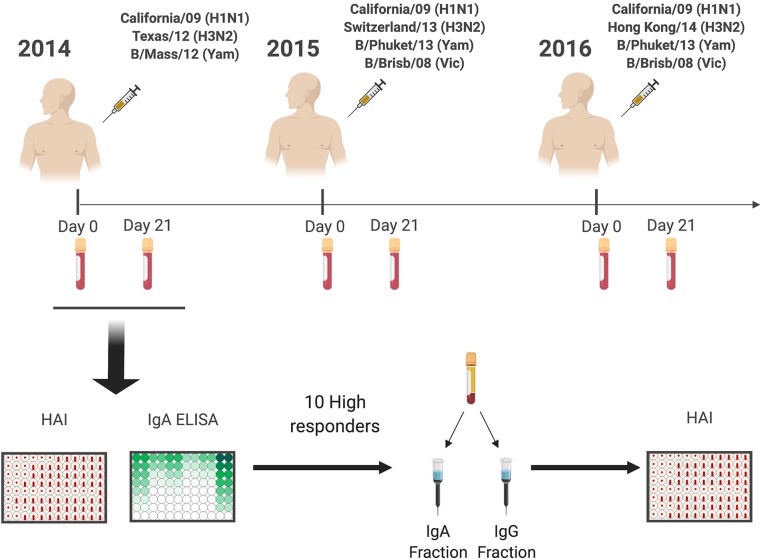
General experimental approach. Healthy subjects (18–35 y.o. and 65–85 y.o.) were vaccinated with standard of care inactivated influenza vaccine for three consecutive years (2014–2016), and serum samples were collected prior (Day 0) and post vaccination (Day 21–28). Serum samples were tested for receptor blocking activity by HAI, or for HA-specific IgA and IgG antibody levels by ELISA. Ten responders with the highest IgA responses were selected for IgA and IgG purification by affinity chromatography and purified fractions used to determine IgA and IgG specific HAI activity.

Eligible volunteers between the ages of 18–35 and 65–85 years old (y.o.), who had not yet received the seasonal influenza vaccine, were enrolled beginning in September of each year, from 2014 to 2016. All vaccine formulations are based on World Health Organization recommendations for the Northern Hemisphere influenza seasons beginning in the Fall (Figure 1), and as such, all vaccinations and sample collections occurred each year between September 1st and December 15th. Influenza virus did not circulate widely in the community during the time periods that the volunteers participated, and as such, participants were not monitored for influenza virus infection during that time-period; they were, however, asked during each visit if they had flu symptoms, and those who did were excluded from the study. Volunteers were recruited at medical facilities in two sites: Pittsburgh, Pennsylvania and Stuart, Florida. All were enrolled with written, informed consent. Exclusion criteria included documented contraindications to Guillain-Barré syndrome, dementia or Alzheimer's disease, allergies to eggs or egg products, estimated life expectancy <2 years, medical treatment causing or diagnosis of an immunocompromising condition, or concurrent participation in another influenza vaccine research study. These two cohorts spanned for 4 years from 2013 to 2016 (21, 22). However, for this study only the 59 (24 young and 35 elderly) repeatedly vaccinated subjects from 2014 to 2016 were selected to characterize the serological IgA response to the vaccine. Serological hemagglutination inhibition (HAI) responses from recurrent vaccinated subjects were similar to matching age groups of the original cohorts (data not shown). Blood (70–90 mL) was collected from each subject at the time of vaccination (D0) and 21–28 days (D21) post-vaccination. Blood samples were processed for sera and peripheral blood mononuclear cells (PBMC). For PBMC isolation, blood was collected in CPT tubes (Becton, Dickinson and Company, Franklin Lakes, NJ, USA) at D0 and D21. These samples were processed immediately, within 1–24 h of collection, and stored at −150°C for future analysis. Sera was collected in SST tubes (Becton, Dickinson and Company) and processed within 24–48 h, storing at 4°C until separated and aliquoted for long-term storage at −30°C. These serum samples were tested for the ability to mediate HAI and HA-specific IgA antibodies against the matching and past vaccine strains. Throughout the study, the H1N1 strain (A/California/7/2009) in the vaccine remained constant for three seasons, whereas the H3N2 (A/Texas/50/2012 in 2014, A/Switzerland/9715293/2013 in 2015, and A/Hong Kong/4801/2014 in 2016) vaccine strains were updated and changed each season.

### Viruses and HA Antigens

Influenza viruses were obtained through the Influenza Reagents Resource (IRR), BEI Resources, the Centers for Disease Control and Prevention (CDC), or were provided by Sanofi Pasteur (Cambridge, MA, USA) and Virapur, LLC (San Diego, CA, USA). Viruses were passaged once in the same growth conditions as they were received, in 10-day old embryonated, specific pathogen-free (SPF) chicken eggs per the protocol provided by the WHO. Due to low influenza B virus sensitivity in the HAI test, viruses underwent ether-treatment as recommended by the Influenza Division of the CDC in order to increase assay sensitivity and more reliable detection of HAI rises following influenza B vaccination (27). Ether-extracted split viruses were created from freshly harvested allantoic fluid and from previously frozen virus lots, prior to HA and HAI assays, as previously described (22). Briefly, viruses were mixed at a 1:1 ratio with anhydrous diethyl ether (ACROS Organics/Fisher Scientific, Pittsburgh, PA, USA) for ≧4 h, with stirring. Following phase separation, ether was evaporated under a fume hood. Titrations before and after ether treatment were performed with turkey erythrocytes and virus was standardized to 8 HAU/50 μL for use in HAI assays. The virus used in this study matched the four vaccine strains included in the 2016 commercially licensed split-virion (IIV) Fluzone™ (Sanofi Pasteur, Swiftwater, PA, USA) influenza vaccine.

### Recombinant HA Proteins

Full-length HA proteins were developed for each of the Fluzone^TM^ influenza A vaccine components: A/California/7/2009 (H1N1), A/Texas/50/2012 (H3N2), A/Switzerland/9715293/ 2013 (H3N2), A/Hong Kong/4801/2014 (H3N2) as well as past H1N1 and H3N2 vaccine strains (Table S1). A chimeric HA protein was generated by cloning the head portion of A/mallard/Sweden/81/2002 (A/H6N2) on top of A/California/07/2009 (A/H1N1_pan_) stem region (cH6/1) (28, 29). Correct stem conformation was validated by enzyme-linked immunosorbent assay (ELISA) using FI6, CR6261 (Creative Biolabs, Shirley, NY, USA) and C179 (Takara Bio, Mountain View, CA, USA) stem-directed monoclonal antibodies (29, 30). Recombinant HA (rHA) proteins representing the wild type and chimeric amino acid sequence were expressed in EXPI293 cells and purified via a C-terminal histidine tag on HisTrap excel nickel-affinity chromatography columns (GE Healthcare Life Sciences, Marlborough, MA, USA) as previously described (29, 30). Purified rHA proteins were dialyzed against PBS, total protein concentration adjusted to ~1 mg/mL after bicinchonic acid (BCA) assay (Thermo Fisher Scientific, Waltham, MA, USA) estimation and purity checked by sodium dodecyl sulfate-polyacrylamide gel electrophoresis (SDS-PAGE).

### HA-Specific IgA Enzyme Linked Immunosorbent Assay (ELISA)

Immulon® 4HBX plates (Thermo Fisher Scientific, Waltham, MA, USA) were coated with 50 ng/well of rHA in carbonate buffer (pH 9.4) with 250 ng/mL bovine serum albumin (BSA) for ~16 h at 4°C in humidified chambers. Plates were blocked with blocking buffer (2% BSA, 1% gelatin in PBS/0.05%Tween20) at 37°C for 2 h. D0 and D21 serum samples were initially diluted 1:50 and then further 2-fold serially diluted in blocking buffer to generate 7-point binding curves. Serially diluted serum samples were added to the assay plate in duplicate and incubated ~16 h overnight at 4°C in humidified chambers. Plates were washed 4 times with phosphate buffered saline (PBS) and HA-specific IgA detected using horseradish peroxidase (HRP)-conjugated goat anti-human IgG (Southern Biotech, Birmingham, AL, USA) at a 1:4,000 dilution and incubated for 2 h at 37°C. Plates were then washed 5 times with PBS prior to development with 100 μL of 0.1% 2,2'-azino-bis(3-ethylbenzothiazoline-6-sulphonic acid) (ABTS) solution with 0.05% H_2_O_2_ for 20 min at 37°C. The reaction was terminated with 1% (w/v) SDS. Colorimetric absorbance at 414 nm was measured using a PowerWaveXS (Biotek, Winooski, VT, USA) plate reader. HA-specific IgA equivalent concentration was calculated based on an 8-point standard curve (2–250 ng/mL) generated using a human IgA reference protein (Athens Research and Technology, Athens, GA, USA). Cumulative HA binding was calculated by adding the IgA-equivalent of both IAV vaccine components (H1+H3).

### Hemagglutination-Inhibition (HAI) Assay

The hemagglutination inhibition assay was used to assess functional antibodies to the H1N1 HA able to inhibit agglutination of turkey erythrocytes as previously described (21, 22). The protocols were adapted from the WHO laboratory influenza surveillance manual (16). To inactivate non-specific inhibitors, sera were treated with receptor-destroying enzyme (RDE) (Denka Seiken, Co., Japan) prior to being tested. Briefly, three parts of RDE were added to one part of serum and incubated overnight at 37°C. RDE was inactivated by incubation at 56°C for 30–45 min and then cooled to RT before diluting with 1X PBS or 0.85% NaCl to a final serum dilution of 1:10. RDE-treated sera was serially diluted in PBS 2-fold across v-bottom microtiter plates (Greiner bio-one, Monroe, NC, USA). An equal volume of each influenza virus (25 μL), adjusted to a concentration of ~8 HAU/50 μL, was added to each well. The plates were covered and incubated at RT for 20 min, and then erythrocytes (Lampire Biologicals, Pipersville, PA, USA) in PBS were added. Red blood cells were stored at 4°C and used within 72 h of preparation. The plates were mixed by agitation and covered, and the RBCs settled for 30 min at RT. The HAI titer was determined by the reciprocal dilution of the last well that contained non-agglutinated RBCs. Positive and negative serum controls were included in each plate. Seroprotection was defined as HAI titer >1:40 and seroconversion as a 4-fold increase in titer compared to baseline resulting in a titer of >1:40, as per the WHO and European Committee for Medicinal Products to evaluate influenza vaccines (16). Subjects were considered seronegative with a titer <1:40.

### Purification of IgA and IgG Antibodies

Ten subjects (five donors 18–35 y.o. and five donors 65–85 y.o.) with the highest HA-specific IgA titers post-vaccination were selected for isotype-specific antibody fractionation (Figure 1). IgA1 and IgG antibodies were purified using jacalin agarose (Thermo Fisher Scientific, Waltham, MA, USA) and lectin beads (Thermo Fisher Scientific, Waltham, MA, USA) affinity chromatography, respectively. In brief, D0 and D21 collected sera (200 μL) were diluted 1:1 with PBS supplemented with 0.02% sodium azide (PBSA) and added to a jacalin bead gravity column. The column was then washed with PBSA until the solution had an optical density (O.D.) value of zero at 280 nm wavelength. The beads were then eluted in 2 mL fractions with α-d-galactose (0.1 M) (Sigma-Aldrich, St. Louis, MO, USA) until the same optical density was reached. The column was regenerated with PBSA (20 mL). The remaining flow through, as well as the wash collection was placed in a Protein G column (Thermo Fisher Scientific, Waltham, MA, USA) (5 mL) and washed with PBSA (75 mL). After baseline, the sample was eluted with 12 mL of 0.1 M glycine (pH 2.5) and the eluate was collected in 2 mL fractions. Samples were neutralized with 1.5 M Tris (200 μL at pH 8.5) and all fractions were dialyzed three times with PBSA and concentrated using 30k Spin-X UF tubes (Nunc, Corning, Thermo Fisher Scientific, Waltham, MA, USA) to ≅250 μL. Concentrator filter was then washed with 75 μL of PBSA and added to the final collection tube. Protein content was estimated by BCA assay and adjusted to 0.5–1 mg/mL concentration. Purity and yield were determined by ELISA; IgA and IgG fractions were 90–97% pure with a 97–99% yield (Figure S1). Purification of Ig isotypes had no impact on the ratio of HA-specific IgA or IgG antibodies (data not shown).

### Total IgA and IgG ELISA

Costar ELISA plates (Thermo Fisher Scientific, Waltham, MA, USA) were coated with 2 μg/mL goat anti-human Ig UNLB (Southern Biotech, Birmingham, Alabama) and incubated overnight at 4°C in a humidifying container. Plates were blocked with blocking buffer at 37°C for 2 h. D0 and D21 serum samples were initially diluted 1:50 for IgA and 1:500 for IgG, while —the corresponding IgA and IgG purified fractions were initially diluted to 1:1,500 and 1:50,000, respectively. All samples were then further 2-fold serially diluted in blocking buffer to generate 7-point binding curves and incubated overnight at 4°C. Plates were washed 4 times and detection and development performed as described above. Total IgA and IgG concentration was calculated based on an 8-point standard curve (2–250 ng/ml) generated using a human IgA or human IgG reference protein (Athens Research and Technology, Athens, GA, USA). Yield was calculated as $\frac{IgA\;recovered}{IgA\;input}\;X\;100$ and purity was calculated as $\frac{IgA\;in\;IgA\;fraction}{\left(IgA\;+\;IgG\right)\;in\;IgA\;fraction}\;X\;100$.

### Flow Cytometry

Human PBMC (~5 × 10^6^ live cells) were stained on ice for 30 min with 100 μL of staining buffer [PBS/2% fetal bovine serum (FBS)]. Human PBMC were first treated with Fc receptor blocking solution (Biolegend, Dedham, MA, USA) then stained for 30 min on ice using titrated quantities of fluorescently conjugated monoclonal antibodies (Table S1). After completion of surface labeling, human PBMC were washed extensively with staining buffer prior to fixation with 1.6% paraformaldehyde in staining buffer for 15 min at RT. Following fixation, cells were pelleted by centrifugation at 400 x *g* for 5 min, resuspended in staining buffer and maintained at 4°C protected from light until acquisition. Data acquisition was performed using the BD FACSARIA Fusion and analysis performed using FlowJo (FlowJo LLC, Ashland, OR, USA). Compensation values were established prior to acquisition using appropriate single stain controls. Memory B cells were defined as CD3/CD14^neg^ CD19^+^, CD27^+^, IgD^−^ cells as previously described (31, 32).

### Statistical Analysis

Statistical significance between groups was calculated using one-way ANOVA Friedman test and Dunns multiple comparisons. Values were considered significant for *p* < 0.05. Unless otherwise stated, data is presented from at least three independent experiments.

Percentage of HA binding to each vaccine strain was calculated from the cumulative IgG or IgA binding to the IAV vaccine components for each subject individually (H1+H3).

Significant subtype immunodominance was determined as previously described (19). In brief, significant immunodominance in a group was calculated by One-sample Wilcoxon Signed rank test (%HA≠50) and 1-way ANOVA Friedman test and Dunn's multiple comparisons (H1≠H3). Statistical significance (*p* < 0.05) must be reached in both tests and the highest *p*-value is represented. Differences between pre- and post-vaccination were calculated by one-way ANOVA multiple comparisons.

Significant immunodominance for each donor was assessed by two independent multiple *t*-test one per row. Replicate readings (n ≧ 6) of HA-specific IgG or IgA and percentage of HA binding were tested for significant differences between vaccine components (H1 ≠ H3 & %H1 ≠ %H3 ≠ 50%). Statistical significance (*p* < 0.05) must be reached in both tests. Subjects with readings below the limit of detection were excluded from the analysis.

Intra- and inter-assay significant relationships were determined by Pearson correlation analysis. All statistical analysis was performed using the GraphPad Prism V.8.3.0 software (San Diego, CA).

## Results

### Recurrent IIV Vaccination Induces H1N1 Reactive IgA Antibodies in Young and Elderly Subjects

**Figure 2:**
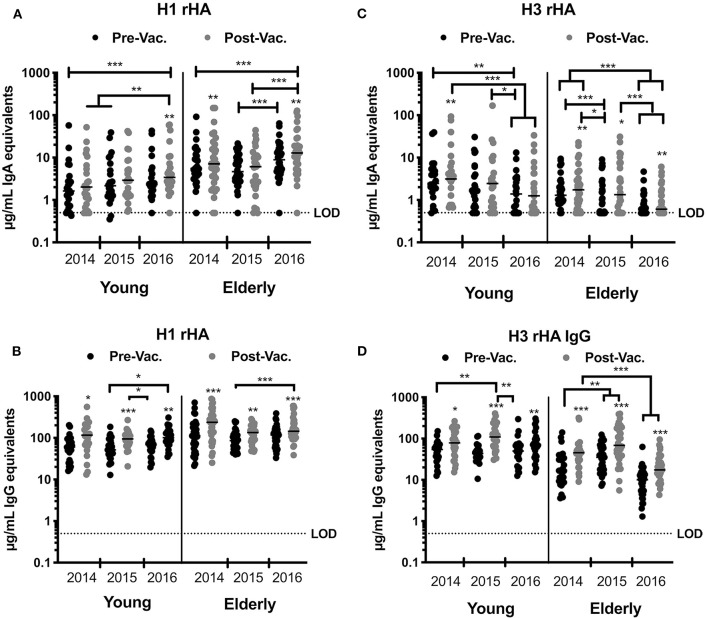
IIV recurrent vaccination induces H1N1-specific IgA and IgG antibodies in young and elderly subjects. HA-specific IgA and IgG levels in the serum of young and elderly donors was measured by ELISA. **(A,B)** Serum samples from young adults and elderly subjects collected prior and 28 days post-vaccination for three consecutive years were tested for anti-HA specific IgA **(A)** or IgG **(B)** antibodies against the H1N1 vaccine strain (CA/09) rHA. **(C,D)** Serum samples from young adults and elderly subjects collected prior and 28 days post-vaccination for three consecutive years were tested for anti-HA specific IgA **(C)** or IgG **(D)** antibodies against H3N2 vaccine strains rHA (TX/12 in 2014, Switz/13 in 2015, and HK/14 in 2016). Box and whisker plots show the median with upper and lower quartile of the μg/mL IgA or IgG equivalent based on a human reference serum standard. *p < 0.05, **p < 0.01, ***p < 0.001.

Vaccination with split-inactivated influenza vaccines (IIV) induces HA-specific IgG antibodies (21). However, the impact of recurrent consecutive IIV vaccination on the serological IgA antibody response has not been thoroughly investigated. To better understand the serological response to recurrent IIV vaccination with antigenically similar vaccine strains, the serological IgA antibody titers were quantified against the H1N1 HA vaccine component (A/California/07/09) in young and elderly subjects vaccinated over three consecutive northern hemisphere influenza seasons (2014 to 2016) (Figure 2A). Elderly subjects (age 65–85 y.o.) had a significant rise in specific anti-HA IgA antibody titers to the H1N1 HA after vaccination in 2014 and 2016, but not in 2015. In young subjects (18–34 y.o.), despite a consistent trend for increased IgA antibody titers against H1N1 HA vaccine component following vaccination, IIV vaccination did not significantly increase these titers until the 2016 season (Figure 2A). Nonetheless, recurrent vaccination over three consecutive years with IIV significantly increased the anti-HA IgA antibody titers in both elderly and young subjects ($Geo\;Mean\;Dif\;({H1}_{16_{D21}}-{H1}_{14_{D0}})$ 7.3 and 1.763 μg/mL, respectively). Interestingly, elderly subjects had significantly higher titer of anti-HA H1N1-reactive IgA antibodies both prior to- and post-vaccination in 2014 and 2016, but not in 2015. IgG antibodies against the H1N1 HA component of the vaccine had a similar trend to IgA response (Figure 2B). From 2014 to 2016, the IgA and IgG antibody titers were comparable prior to vaccination, which indicates a transient rise even after recurrent vaccination with the same vaccine strain (Figures 2A,B).

### Reduced Anti-HA Serological IgG and IgA Antibody Titers to the H3N2 Vaccine Component Following Recurrent Vaccination With Antigenically Different Vaccine Strains

Between 2014 and 2016, the recommended H3N2 component in the seasonal influenza vaccine was updated each season due to viral antigenic drift (33, 34). To understand the serological response to recurrent IIV vaccination with 3 antigenically different H3N2 vaccine strains, the levels of serological IgA antibodies that bound to the HA from A/Texas/50/2012 (TX/12), A/Switzerland/9715293/ 2013 (Switz/13), and A/Hong Kong/4108/2014 (HK/14) were determined in young and elderly subjects that were vaccinated over these three consecutive influenza seasons (Figure 2C). In 2015 and 2016, IIV vaccination had no significant impact on H3 HA-specific IgA antibody titers in young subjects, but there was a trend for decreased titers across years ($Geo\;Mean\;Dif\;\left({H3}_{16_{D21}}-{H3}_{14_{D21}}\right)\;=-$1.54 μg/mL). Vaccinated elderly subjects had a significant increase in the H3 HA-specific IgA antibodies each season (*p* < 0.001). However, there were no significant differences in these IgA titers prior to- or post-vaccination in both the elderly and young subjects. Furthermore, elderly subjects had a significant decrease in H3 HA-specific IgA titers from 2014 to 2016 ($Geo\;Mean\;Dif\;\left({H3}_{16_{D21}}-{H3}_{14_{D21}}\right)$ = −1.02 μg/mL). In contrast, there was a significant increase during each season in serological anti-H3 HA-specific IgG antibodies following vaccination in both young and elderly subjects (Figure 2D). Similar to H3 HA-specific IgA titers, there was a decrease in the IgG titers in elderly subjects in 2016, as well as a decrease in the magnitude of the IgG response following vaccination, ($Geo\;Mean\;Dif\;\left({H3}_{D21}-{H3}_{D0}\right)$ = 20.94; 28.57, and 6.35 μg/mL in 2014, 2015, and 2016, respectively) (Figures 2C,D).

### Serological H1 HA-Specific IgA Titers Positively Correlate With IgG Titers

Influenza vaccination has similar impact on serum IgA and IgG antibody titers (Figure 2). To understand the relationship between the HA-specific IgA and IgG antibodies, the inter-assay Pearson correlations for each timepoint were calculated (Tables 1, 2). H1-specific IgA titers are positively correlated with IgG titers at all timepoints, except for D21 post-vaccination in 2015 (*p* = 0.11) (Table 2). In contrast, H3 HA-specific IgA titers only correlate with serum IgG titers in 2014, but not in 2015 or 2016 (Table 2).

**Table 1:** Inter-assay Pearson correlations for H1N1 vaccine strains.

**Timepoint**	**IgA/IgG (μg/ml)**	**IgA(μg/ml)/ Log2(HAI Titer)**
14_D0	0.53^***^	ns
14_D21	0.62^***^	ns
15_D0	0.36^**^	−0.32^*^
15_D21	ns	ns
16_D0	0.57^***^	−0.37^*^
16_D21	0.51 ^***^	ns

n = 59 (24 young + 35 Elderly);

* p < 0.05,

** p < 0.001,

*** *p < 0.001*.

**Table 2:** Inter-assay Pearson correlations for H3N2 vaccine strains.

**IgA (μg/ml)**	**IgA/IgG (μg/ml)**	**IgA(μg/ml)/ Log2HAI Titer**
14_D0	0.39^**^	Ns
14_D21	0.50^***^	Ns
15_D0	0.26^*^	ns
15_D21	0.32^*^	0.33^*^
16_D0	0.36^**^	ns
16_D21	0.42^**^	ns

n = 59 (24 young + 35 Elderly);

* p < 0.05,

** p < 0.01,

*** *p < 0.001*.

Following IIV vaccination, changes in the antibody titers are generally assessed by the HAI assay, thus the inter-assay Pearson correlations between HA-specific IgA and serological HAI activity against the H1N1 and H3N2 vaccine strains at each timepoint were calculated (Tables 1, 2). Longitudinally, HA-specific IgA antibody titers do not positively correlate with HAI titers against the H1 or H3 vaccine components at most timepoints tested. The only exception was in 2015 following vaccination, where the H3 HA-specific IgA titers significantly correlated with serum HAI titer against the Switz/13 H3N2 virus strain (*r* = 0.33, *p* = 0.018). Surprisingly, the H1N1 HA-specific antibodies at D0 in 2015 and 2016 were negatively correlated with the serological HAI activity against the H1N1 CA/09 virus (Table 1).

### Pre-Existing HA-Specific IgA Titers Positively Correlated With Post-Vaccination Titers

To understand the relationship between pre- and post-vaccination antibody titers, we calculated intra-assay Pearson correlations between all timepoints (Tables 3–6). Overall, pre-existing HA-specific IgA antibody titers positively correlated with HA-specific IgA antibody titers after vaccination for both H1 and H3 HA vaccine components (Tables 3, 4). In contrast, in 2014, the H3N2 HA-specific IgG titers post-vaccination did not correlate with H3N2 HA-specific IgG titers in 2015 (Tables 5, 6).

**Table 3:** Intra-assay Pearson correlations for H1N1 vaccine strains.

**IgA (μg/ml)**	**14_D0**	**14_D21**	**15_D0**	**15_D21**	**16_D0**
14_D21	0.80				
15_D0	0.54	0.49			
15_D21	0.44	0.50	0.70		
16_D0	0.69	0.59	0.68	0.48	
16_D21	0.75	0.68	0.61	0.53	0.89

**Table 4:** Intra-assay Pearson correlations for H3N2 vaccine strains.

**IgA (μg/ml)**	**14_D0**	**14_D21**	**15_D0**	**15_D21**	**16_D0**
14_D21	0.77				
15_D0	0.81	0.72			
15_D21	0.60	0.57	0.66		
16_D0	0.73	0.67	0.76	0.70	
16_D21	0.71	0.66	0.69	0.79	0.81

**Table 5:** Intra-assay Pearson correlations for H1N1 vaccine strains.

**IgG (μg/ml)**	**14_D0**	**14_D21**	**15_D0**	**15_D21**	**16_D0**
14_D21	0.76				
15_D0	0.54	0.28			
15_D21	0.38	0.22	0.75		
16_D0	0.56	0.39	0.66	0.67	
16_D21	0.48	0.29	0.56	0.55	0.75

*N = 59, 24 young and 35 elderly; All values are significantly different from 0, p < 0.05, unless if highlighted in red*.

**Table 6:** Intra-assay Pearson correlations for H3N2 vaccine strains.

**IgG (μg/ml)**	**14_D0**	**14_D21**	**15_D0**	**15_D21**	**16_D0**
14_D21	0.66				
15_D0	0.57	0.27			
15_D21	0.38	0.22	0.49		
16_D0	0.66	0.40	0.64	0.48	
16_D21	0.56	0.35	0.50	0.53	0.72

*N = 59, 24 young and 35 elderly; All values are significantly different from 0, p < 0.05, unless if highlighted in red*.

### Elderly Subjects Have a Highly Immunodominant Anti-HA IgA Response to the H1N1 Vaccine Component

**Figure 3:**
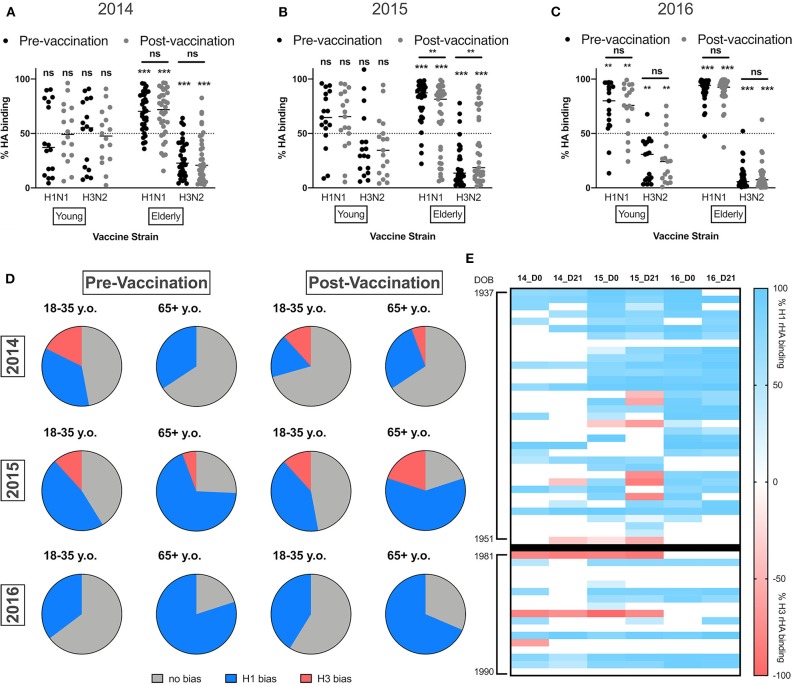
Elderly subjects have significantly immunodominant response to the H1N1 vaccine component. **(A–C)** Percentage of IgA HA-binding to the H1N1 or the H3N2 vaccine strain calculated as described in the M&M section in young adults and elderly subjects vaccinated in 2014 **(A)** 2015 **(B)** or 2016 **(C)**. **(D)** Frequency of young adults and elderly subjects with a significant immunodominant IgA response toward the H1N1 or H3N2 vaccine strains pre- and post-vaccination. **(E)** Heatmap for the percentage of immunodominant response to each IAV vaccine strain from 2014 to 2016. Donors are represented as rows organized by date of birth (DOB). Blue indicates significant immunodominance of the H1N1 vaccine strain, red represents significant immunodominance of the H3N2 vaccine strain, and white shows balanced responses. **p < 0.01, ***p < 0.001.

Multivalent vaccines assume equal immunogenicity of the vaccine components to induce a balanced immune response to each vaccine strain (35). However, more often, there is a subtype immunodominance to one or more components following influenza vaccination (19). To understand the impact of influenza vaccination on the balance of the serological response to influenza A virus, the percentage of HA binding to H1 and H3 HA vaccine components was quantified. Ideally, in the absence of subtype immunodominance, ~50% of total anti-HA antibodies would bind to each of the two influenza A vaccine HA components. Prior to vaccination in 2014, young adults did not appear to have an immunodominance to either of the IAV vaccine strains (Figure 3A). In contrast, elderly subjects had a significant subtype immunodominance toward the H1 HA vaccine component (mean difference to 50% = 19.3% ± 1.7, *p* < 0.001). Vaccination with TX/12 or HK/14 in 2014 and 2016 did not alter pre-existing subtype immunodominance, while in elderly subjects, vaccination with Switz/13 H3N2 vaccine strain in 2015 resulted in a slight decrease in the H1 HA immunodominance (mean difference to 50% = 16.3% ± 4.6, *p* = 0.002). Interestingly, consecutive vaccinations with the same H1N1 vaccine strain, while the H3N2 vaccine component was changed each season, resulted in significant H1 HA subtype immunodominance in young adults during the 2016 season (mean dif. to 50% = 40% ± 11.39, *p* < 0.001).

Finally, the frequency of young and elderly subjects with significant subtype immunodominance (significant differences in the response to one of the IAV vaccine components) was calculated. Despite of similar frequencies of individuals with a balanced antibody response against both IAV vaccine strains, an equal number of young adults are immunodominant toward H1 or H3 HA components, while elderly subjects favor the H1 HA vaccine component (Figure 3D).

### Split-Inactivated Influenza Vaccination Does Not Induce IgA to Past Seasonal IAV Vaccine Strains

**Figure 4:**
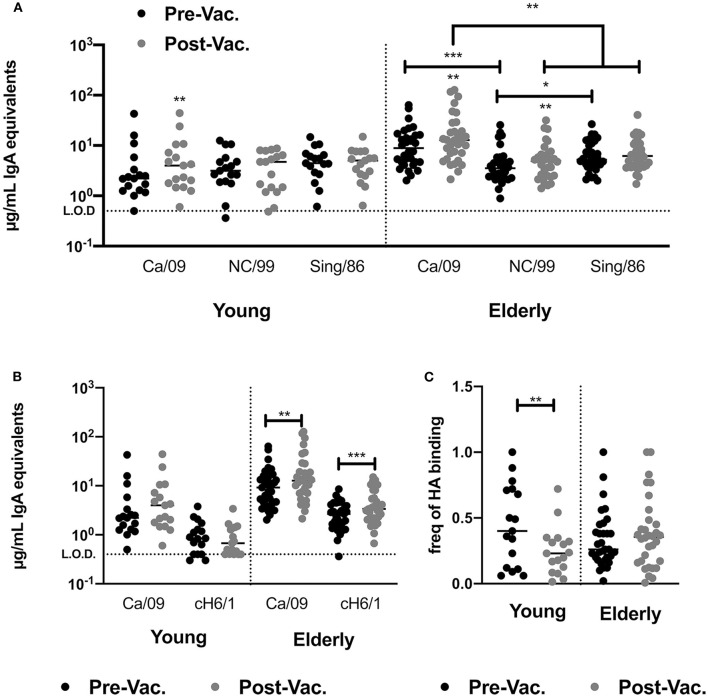
IIV vaccination does not recall broadly reactive IgA antibodies in young and elderly subjects. **(A)** Levels of HA-reactive IgA antibodies against rHAs from three H1N1 influenza viruses (CA/09, NC/99 and Sing/86) in the serum of young adults and elderly subjects prior to and post-vaccination. **(B)** Level of H1 stem-directed IgA antibodies in the serum of young adults and elderly subjects prior to and post-vaccination, measured by ELISA as described in the M&M section. **(C)** Frequency of stem-directed IgA antibodies relative to total rHA binding, in the serum of young adults and elderly subjects prior to and post-vaccination. *p < 0.05, **p < 0.01, ***p < 0.001.

Inactivated influenza vaccines generally recall pre-existing memory B-cells. To understand if subtype immunodominance is the result of high pre-existing cross-reactive H1 HA IgA antibodies, we quantified the levels of serological IgA antibodies against two past H1N1 HA vaccine strains (Sing/86 and NC/99) prior to- and after vaccination in 2016. In young adults, pre-existing titers of CA/09 HA-reactive IgA antibodies in the serum were similar to the titers against two historical vaccine strains, Sing/86 and NC/99. The IIV vaccination did not increase historical H1 HA-reactive IgA antibodies. In contrast, elderly subjects had significantly higher IgA antibody titers against the CA/09 HA vaccine strains than against the two historical strains and vaccination significantly induced NC/99 HA-reactive IgA antibodies (Figure 4A).

### Split-Inactivated Influenza Vaccination Does Not Induce HA-Stem IgA Antibodies

Split-inactivated influenza vaccinations induce stem-directed IgG memory responses. Measuring peripheral antigen-specific IgA memory responses is technically challenging, thus the impact of IIV on the IgA serological response against the H1 HA stem by ELISA was measured using a chimeric rHA (cH6/1)(29, 30, 36). IIV vaccination does not induce stem-directed IgA antibodies against the H1 or H3 HA vaccine components (Figure 4B). However, elderly subjects had a significant increase in stem-directed IgA antibodies compared to young adults, who had a significantly lower percentage of stem-directed IgA antibodies, compared to head-directed antibodies, following vaccination (Figure 4C).

### IgA Antibodies Have Receptor Blocking Activity

To understand the significance of HA-specific IgA antibodies to prevent receptor binding, IgA and IgG antibodies were purified from the serum of 10 vaccinated donors with high post-vaccination HA-specific antibody titers. IIV vaccination significantly increased the receptor blocking antibodies against the H1N1 and H3N2 vaccine strains, as reflected by a decrease in serological Ig minimal effective concentration (MEC) required to prevent viral hemagglutination activity. Similarly, after normalizing the serum IgG and IgA antibody titers, vaccination significantly decreased the MEC required for HAI activity against the H1N1 and H3N2 vaccine strains. Purified IgG and IgA antibodies had similar MEC as the total serum Ig HAI activity against both vaccine strains, however, this was not impacted by vaccination. After normalizing to HA-specific antibody titers, there were no significant differences in HAI activity between IgG and IgA antibodies, prior to- or post-vaccination (Tables 7, 8).

**Table 7:** Minimal Effective Concentration for HAI activity.

**H1N1 strain**	**Pre vaccination**		**Post vaccination**	
	**Geo Mean (ng/ml)**	**(±SD)**	**Geo Mean (ng/ml)**	**(±SD)**
Total Ig Serum	107.6^a^	3.34	28.7^b^^,^^c^	2.30
IgG Serum	90.93^a^	3.26	20.85^b^^,^^c^	2.34
IgA serum	16.28^b^	3.75	7.26^d^	2.46
Purified IgG	52.07^a^^,^^c^	2.48	70.71^a^^,^^c^	1.63
Purified IgA	85.59^a^	1.96	58.12^a^^,^^c^	3.07
HA-specific purified IgG	0.91^d^	3.58	2.63^d^	5.21
HA-specific purified IgA	1.85^d^	4.15	1.93^d^	5.20

*N = 10, 5 young (18–35 y.o.), and 5 elderly (>65 y.o.) subjects*.

a−d *Paired two-way ANOVA grouping*.

**Table 8:** Minimal Effective Concentration for HAI activity.

**H3N2 strain**	**Pre vaccination**		**Post vaccination**	
	**Geo Mean (ng/ml)**	**(±SD)**	**Geo Mean**	**(±SD)**
Total Ig Serum	264.9^a^	2.38	30.76^b^^,^^c^	3.27
IgG Serum	223.9^a^	2.32	22.34^b^^,^^c^	3.25
IgA serum	40^b^	2.70	7.79^d^	3.44
Purified IgG	49.16^a^	2.84	44.56^a^^,^^c^	4.94
Purified IgA	99.48^a^	1.82	102.4^a^^,^^c^	1.53
HA-specific purified IgG	0.160^d^	3.33	0.52^d^	0.13
HA-specific purified IgA	0.12^d^	4.88	5.47^d^	4.90

*N = 10, 5 young (18–35 y.o.), and 5 elderly (>65 y.o.) subjects*.

a−d *Paired two-way ANOVA grouping*.

## Discussion

Seasonal influenza infection remains a major public health concern with significant social and economic impact. Just 2 years ago, people living in the northern hemisphere experienced the highest seasonal influenza activity since the last pandemic in 2009. During the 2017–2018 influenza season, the U.S. CDC estimated almost 50 million people fell ill due to influenza virus infection with >20 million seeking medical attention in the U.S. Furthermore, the 2017–2018 season was marked by severe symptomology across all age groups, leading to almost one million hospitalizations and over 70 thousand deaths (CDC influenza burden). The WHO recommends annual vaccination to prevent seasonal influenza infection and transmission. However, the immune response to recurrent vaccination remains poorly understood. Here, the impact of recurrent vaccination over three influenza seasons on the serological IgA response to influenza A vaccine strains was examined in a small cohort of recurrent vaccines.

From October 2014 to March 2017, the U.S. experienced three influenza seasons of low to mild influenza activity. The 2014–2015 and 2016–2017 seasons were dominated by H3N2 influenza viruses with higher infection and hospitalization rates than the 2015–2016 season, which was dominated by H1N1 influenza viruses. Vaccine effectiveness across all ages and against all vaccine strains ranged from 20 to 50% over these three seasons, with its lowest in 2014 against H3N2 influenza viruses (5%) and highest in 2015 against H1N1 influenza viruses (45%). In this report, we observed that recurrent vaccination that included the same H1N1 vaccine strain (CA/09) significantly increased H1N1 HA-specific serological antibody titers (IgA and IgG) in young and elderly subjects. In contrast, consecutive vaccination with newly updated H3N2 vaccine components seemed to hinder H3N2 HA-specific IgA responses in young and elderly subjects (Figure 2).

Vaccine-induced immune responses are traditionally focused on receptor blocking antibodies or total vaccine-reactive IgG titers as measured by HAI or ELISA, but serological changes in vaccine-specific IgA antibodies are generally neglected. Split-inactivated IIV induces receptor blocking antibodies with high HAI activity against the four vaccine strains (21, 22). Moreover, IIV vaccination raises pre-existing serologic HAI activity against past vaccine strains (21). In contrast, IgA responses following vaccination appear highly strain-specific, with little boost in reactivity against previous vaccine strains or the conserved stem portion of the HA protein (Figure 4).

In the search for a truly universal influenza vaccine, the use of a single immunological assay to assess vaccine-elicited protection is limiting. Recent studies highlight the need for broader and better immune correlates of protection, as well as particular vaccine delivery platforms (37–40). Interestingly, H1N1 HA-specific IgA titers in serum are positively correlated with IgG antibodies against the matched strains in young and elderly subjects for the three seasons assessed in this study (Tables 1, 2). Furthermore, despite similar HAI activity from purified IgA and IgG antibody fractions (Table 7), HA-specific IgA antibody titers in the serum do not correlate with serological HAI activity against the corresponding vaccine strain (Tables 1, 2). This is might result from the artificial nature of the HAI assay or be a consequence of the lower IgA antibody levels in the serum as compared to IgG. Similar studies with other neutralization assays should help clarify the contribution of serological IgA to protection during influenza infection. Alternatively, future work focusing on secreted dimeric IgA, the predominant immunoglobulin in the lung and upper respiratory tract mucosa, will assess the correlation of serological IgA antibody levels with mucosal HAI activity in nasal washes or BAL fluid, since IgA polymerization significantly increases viral neutralization and receptor blocking activity (41).

Recent studies have highlighted the complex dynamics of the antibody repertoire from memory B-cell compartment to plasmablasts and serum (24, 42–44), and exposed the importance of a diverse and balanced clonal recall and *de novo* B-cell responses for broad influenza protection (45). Similar studies focusing on the IgA antibody responses will be crucial to understand the overlap of serological and mucosal IgA antibody repertoires.

Influenza virus infection strongly induces HA-specific polymeric IgA antibodies at the nasal mucosa (46). Furthermore, high influenza-specific IgA antibodies are consistently associated with better disease prognosis and decreased viral transmission (46–48), particularly in subjects with low serological HAI activity (49). Moreover, in animal models, a genetically engineered neutralizing IgA antibody conferred sterilizing immunity and prevented transmission to naïve animals, whereas its parental IgG clone could not (50). Overall, this highlights the significance of serological and mucosal IgA responses for protection against influenza infection.

Influenza virus vaccine production has remained largely unaltered for almost half a century. Nonetheless, the past decades were marked by the emergence of multiple alternative vaccine delivery and production platforms, such as live attenuated virus, virus-like particles (VLP), nanoparticles or recombinant proteins (20, 51–53). Not surprisingly, the immunization route and delivery platform can dramatically impact the immune response to the vaccine (54). Intranasally delivered live attenuated influenza vaccines do not increase serological HAI activity, but have a pronounced induction of influenza-specific mucosal IgA antibodies (20). Similarly, VLP-based vaccines that are generally associated with superior immunogenicity than conventional platforms, strongly induce serological IgA antibodies (55, 56). Together these studies highlight the importance of exploring alternative immune correlates, aside from conventional serological HAI or HA-specific IgG titer, particularly when testing new vaccine delivery systems or immunization routes.

The number of class-switched (IgA and IgG) memory B cells and plasmablasts are age-dependent. While IgG_1_ and IgG_3_ memory B cells peak in number prior to adolescence and continuously decrease as a person ages, IgA memory B cells peak at childhood (2-5 y.o.) and again in early adulthood (18–40 y.o.) followed by a slow decrease throughout life (57). Surprisingly, despite the drastic differences in serological IgA and IgG antibody levels, the overall frequency of IgA and IgG memory B cells in peripheral blood is fairly similar (Figure S2) and does not change from young adulthood to old age (57). Furthermore, despite a prominent increase in activated memory B cells following influenza vaccination, the relative proportion of IgA and IgG memory B cells remains the same (Figure S2). In the peripheral blood, plasma cells frequency peaks during infancy (first two years of age) and steadily decreases throughout life (57), unless transiently expanded by an inflammatory stimulus. Nonetheless, the overall percentage of IgA and IgG plasma cells in circulation is strikingly similar and surprisingly stable throughout life (57).

Recently, our group has reported a subdominant serological IgG response to the H3N2 HA vaccine component during the 2016–2017 influenza season relative to the other three vaccine components (19), as a result of an inefficient recall response of pre-existing H3N2 HA-specific memory B cells. In elderly subjects, IgA responses are significantly skewed toward the H1N1 vaccine strain. In contrast, young adults have a skewed response toward either the H1N1 or the H3N2 vaccine strains (Figure 3). This biased response is most likely the result of early-life influenza virus imprinting. The elderly population was born before 1954 when only H1N1 influenza viruses circulated in the population, whereas young adults born between 1985 and 2001 could have been initially exposed to either H1N1 or H3N2 influenza viruses (58). Moreover, the impact of imprinting in the response to influenza virus vaccination is more severe in the elderly population with impaired *de novo* somatic hypermutation and decreased adaptability to new influenza strains (45).

The pursuit for new influenza vaccine candidates may require a much broader immunological profiling then traditional serological HAI, neutralization assays or vaccine-specific IgG titer. Despite of the limited sample size, this study exposes the significance of serological IgA responses during influenza vaccination, but future work should clarify the overlap of IgA and IgG antibody repertoires, as well as the kinetics and longevity of vaccine-induced antibodies in the serum and at the site of infection. A holistic perspective of the immune response to influenza viruses may lead to the development of a truly universal influenza virus vaccine.

## Data Availability Statement

All datasets generated for this study are included in the article/Supplementary Material.

## Ethics Statement

The studies involving human participants were reviewed and approved by University of Georgia IRB. The patients/participants provided their written informed consent to participate in this study.

## Author Contributions

RA and TR conceived and oversaw the study. EC and SA implemented the assays and interpreted the data. RA performed studies and flow cytometry. GS, RA, and TR reviewed and performed statistics on the data and wrote and edited the manuscript and figures.

## Conflict of Interest

TR has research funding from Sanofi Pasteur, Inc. TR was also supported, in part, by the Georgia Research Alliance as an Eminent Scholar. The remaining authors declare that the research was conducted in the absence of any commercial or financial relationships that could be construed as a potential conflict of interest.
