# Supplementary material for: IgA Responses Following Recurrent Influenza Virus Vaccination
Source: Front Immunol. 2020 May 19;11:902. doi: 10.3389/fimmu.2020.00902 (PMC7249748; doi:10.3389/fimmu.2020.00902)
Supplement: Supplementary file 1 [file Data_Sheet_1.docx]

**IgA Antibody response to recurrent influenza vaccination**

**in the young and elderly**

Rodrigo B. Abreu^1^, Emily F. Clutter^1^, Sara Attari^1^, Giuseppe A. Sautto^1^ and Ted M. Ross^1,2#^

^1^Center for Vaccines and Immunology, University of Georgia, Athens, Georgia, USA

^2^Department of Infectious Diseases, University of Georgia, Athens, Georgia, USA

Frontiers in Immunology

Supplementary data

| Tables S1: Antibodies and dilutions | |  |
| --- | --- | --- |
| **Antibody** | **Cat#** | **Dilution** |
| IgD BV421* | 348226 | 1/200 |
| CD20 BV650* | 302334 | 1/50 |
| CD3 FITC* | 300306 | 1/200 |
| CD14 FITC* | 301804 | 1/200 |
| IgA-Pe** | 130-099-108 | 1/100 |
| CD38 Pe-Dazzel* | 303538 | 1/50 |
| CD27 Pe-Cy7* | 124216 | 1/100 |
| IgG-APC* | 410711 | 1/100 |
| CD19 APC-Fire* | 302258 | 1/100 |
| * Purchased from Biolegend  **Purchased from Miltenyi biotec | | |





**Fig S1**: Percentage of total IgA (**A**) and total IgG (**B**) recovered from serum samples after affinity chromatografy (n=20). Purity of IgA1 and IgG fractions shown as percetage of IgA (**C**) or percentage of IgG (**D**) of total immunoglobulin in the sample.


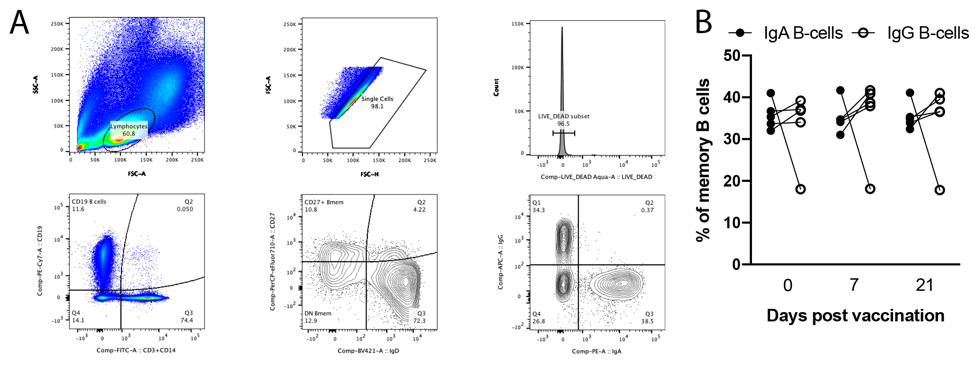


Fig S2: **A**) Representative flow cytometry gating strategy for quantification of IgA and IgG memory B cells. **B**) Summary data of the percentage of IgA and IgG B-cells of memory B cells (CD3/CD14^-^, CD19^+^, CD27^+^, IgD^-^) in 4 donors prior to, 7 days and 21 days post-vaccination, measured as represented in A.
